# From Traditional Medicinal Plant to Modern Pharmacotherapy: A Comprehensive Review of the Bioactive Compounds and Health Applications of *Eucommia ulmoides*

**DOI:** 10.3390/nu18020234

**Published:** 2026-01-12

**Authors:** Wanting Xie, Yubo Xiao, Pan Xu, Hui Zheng, Xianping Zeng, Yuhang Wu, Jiani Jiang, Fan Jia, Jianye Yan, Tao Zheng, Yong Yang

**Affiliations:** 1 School of Pharmacy, Hunan University of Chinese Medicine, Changsha 410208, China; 19974216381@163.com (W.X.); xp589611@163.com (P.X.); huizheng0104@163.com (H.Z.); 14739716868@163.com (X.Z.); 15387030919@163.com (Y.W.); jiangjiani0320@163.com (J.J.); jiafan1227@163.com (F.J.); yanjianye201@126.com (J.Y.); 2 Hunan Provincial Key Laboratory for Synthetic Biology of Traditional Chinese Medicine, School of Medical Laboratory Science, Hunan University of Medicine, Huaihua 418000, China; yuboxiaohn@126.com

**Keywords:** *Eucommia ulmoides*, bioactive compounds, pharmacological effects, health functions, comprehensive utilization

## Abstract

*Eucommia ulmoides* Oliv. (*E. ulmoides*), an endemic tree species in China, holds significant value in traditional Chinese medicine industry and health food. The plant is rich in diverse bioactive compounds, including lignans, iridoids, flavonoids, polysaccharides, *E. ulmoides* gum, amino acids, and minerals. These components contribute to a range of pharmacological activities such as anti-inflammatory, antioxidant, antihypertensive, immunomodulatory, and bone-protective effects, which support its long-standing traditional use and emerging clinical and adjunctive applications. While current research has predominantly focused on the bark and leaves, other parts, such as flowers, seeds, stems and roots, remain underexplored despite their substantial potential for medicinal and edible applications. Based on the recent literature, this paper systematically summarized the chemical composition, health benefits, and comprehensive utilization of different parts of *E. ulmoides* (bark, leaves, flowers, and seeds), aiming to provide a theoretical foundation for the high-value utilization of the entire plant resources of *E. ulmoides*. As a health-promoting plant resource, *E. ulmoides* has extensive development potential in applications such as health foods, natural medicines, and agricultural inputs. Future research should prioritize elucidating the synergistic mechanisms among different active compounds, advancing technologies for multi-part utilization, and establishing standardized quality evaluation systems to facilitate broader applications in functional foods, pharmaceuticals, and related interdisciplinary fields.

## 1. Introduction

*E. ulmoides*, a perennial deciduous tree belonging to the Eucommiaceae family, is a unique species native to China. *E. ulmoides* is primarily distributed in the central, southwestern, and northwestern regions of China, typically growing in sparse forests at altitudes ranging from 300 to 500 m. With a long history of use in traditional Chinese medicine (TCM), *E. ulmoides* was first documented over 2000 years ago in Shen Nong Ben Cao Jing (Divine Farmer’s Materia Medica), where it was classified as a superior herb with health benefits such as “tonifying essence, strengthening tendons and bones, enhancing mental fortitude, and promoting longevity with prolonged consumption” [1]. In TCM, the dried bark of *E. ulmoides* is used as medicine. It is believed to tonify the liver and kidneys, strengthen bones and muscles, and prevent miscarriage. It is primarily indicated for conditions such as liver and kidney deficiency, lower back and knee pain, weakness of tendons and bones, dizziness, blurred vision, gestational bleeding, and restless fetus [2]. In addition to the bark, its flowers, seeds, and leaves are also widely used in medicinal diets and health supplements. Modern pharmacological studies have revealed that *E. ulmoides* exhibits anticancer and antitumor activities, protects the liver and kidneys, regulates immune and endocrine functions, and promotes bone health [3]. Its major bioactive compounds include lignans, iridoids, flavonoids, and polysaccharides [4].

**Figure 1:**
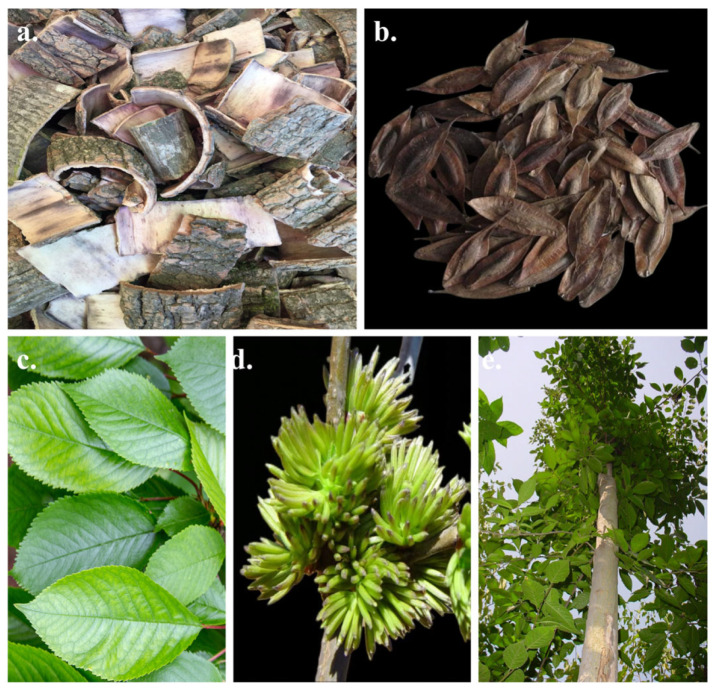
The bark (**a**), the seeds (**b**), the leaves (**c**), the flowers (**d**) and the morphology of plants (**e**) of *E. ulmoides*.

As an important medicinal plant resource, the traditional utilization of *E. ulmoides* has long relied on “felling trees or stripping bark,” which often resulted in slow bark regeneration or even tree death after partial harvesting. The rapidly growing market demand led to over exploitation and severe destruction of *E. ulmoides* resources. In contrast, other parts of the plant, such as leaves, flowers, fruits, and stems, possess strong regenerative capacity and are abundant in supply. Therefore, comprehensive development of *E. ulmoides* resources is of great significance for the sustainable and healthy growth of the *E. ulmoides* industry. Studies have shown that non-traditional medicinal parts, including leaves, male flowers, and fruits, are rich in bioactive constituents also found in the bark, such as lignans, iridoids, flavonoids, polysaccharides, and *E. ulmoides* gum (EUG) [5]. These components demonstrate various functions, including antihypertensive, lipid-lowering, antioxidant, anti-inflammatory, immunomodulatory, and bone maturation-promoting effects [6]. In China, *E. ulmoides* leaves have been included in the catalog of substances that are both food and medicinal herbs, *E. ulmoides* male flowers and *E. ulmoides* seeds oil have been approved as Novel Food Ingredients. These developments signify the expansion of *E. ulmoides* applications from traditional medicine to broader health-related fields. Based on the recent scientific literature, this review systematically summarizes the advances in the chemistry, pharmacological mechanisms, and innovative food applications of *E. ulmoides*. This paper aims to provide a theoretical basis for the high-value, comprehensive utilization of *E. ulmoides* resources within the modern health and food industries (Figure 1). In addition to summarizing the chemical composition and biological activities of different botanical parts, this review attempts to assess the quality and level of available evidence, with particular attention to the translational relevance of preclinical findings to clinical and functional food applications, and highlights potential research gaps that may currently limit clinical validation and industrial utilization.

## 2. Material and Methods

This review was conducted as a narrative literature review based on a structured search strategy. Relevant studies related to *E. ulmoides* were retrieved from PubMed, Web of Science, CNKI, Google Scholar, and the Pharmacopoeia of the People’s Republic of China. The literature search covered publications from January 1995 to June 2025 using the keywords “Eucommia ulmoides”, “botany”, “traditional uses”, “chemical constituents”, “bioactive compounds”, “pharmacological activities”, “health functions”, and “comprehensive utilization”, alone or in combination. Original research articles and authoritative reviews published in peer-reviewed journals in English or Chinese that reported on the chemical composition, pharmacological effects, or health-related applications of *E. ulmoides* and its different botanical parts were included, whereas duplicate publications, studies lacking clear experimental design or outcome description, and non-scientific materials such as conference abstracts or editorials were excluded. Retrieved records were screened based on titles and abstracts, followed by full-text evaluation, and the eligible literature was qualitatively analyzed and synthesized to provide an integrated overview of the chemical basis, pharmacological mechanisms, and utilization potential of *E. ulmoides*.

## 3. Compositional Basis of *E. ulmoides* Resources

**Figure 2:**
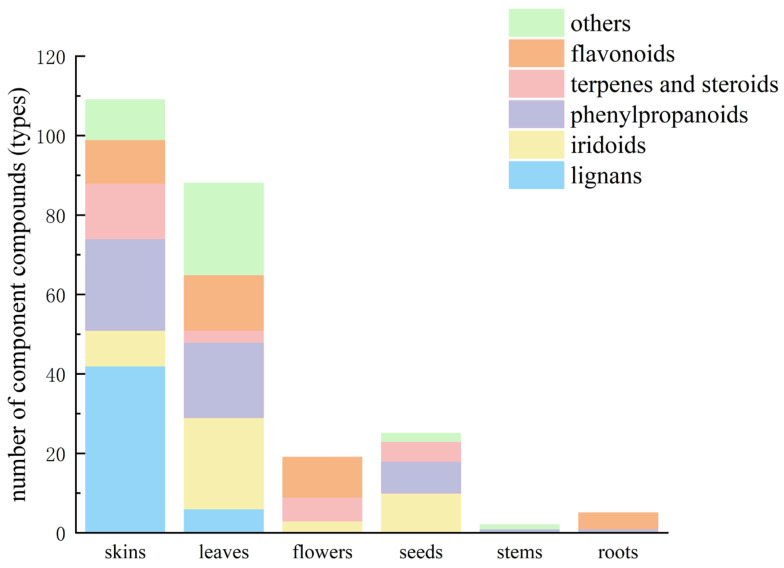
Distribution of components and compounds of resources from different parts of *E. ulmoides*.

*E.ulmoides* contained diverse phytochemicals including lignans, iridoids, phenylpropanoids, flavonoids, terpenoids, steroids, polysaccharides, amino acids, vitamins, minerals, and substantial EUG, with compositional distribution across botanical parts illustrated in Figure 2 [4]. Traditional analytical efforts predominantly focused on the medicinal bark. However, subsequent investigations have identified rich phytochemical profiles in leaves, flowers, seeds and stems. Iridoids, phenolics, and flavonoids constitute major constituents in leaves, while male flowers are primarily characterized by flavonoid components. The highest lignan and iridoid concentrations were detected in the bark, whereas leaves exhibit peak levels of flavonoids and chlorogenic acid. EUG content is maximal in seeds. Iridoid, lignan, and phenylpropanoid concentrations correlate with plant parts, while flavonoid content associates with tree age [7]. At the molecular level, we systematically summarize how these compounds exert their pharmacological activities, offering a scientific basis for future drug development and clinical applications.

### 3.1. Lignans

**Figure 3:**
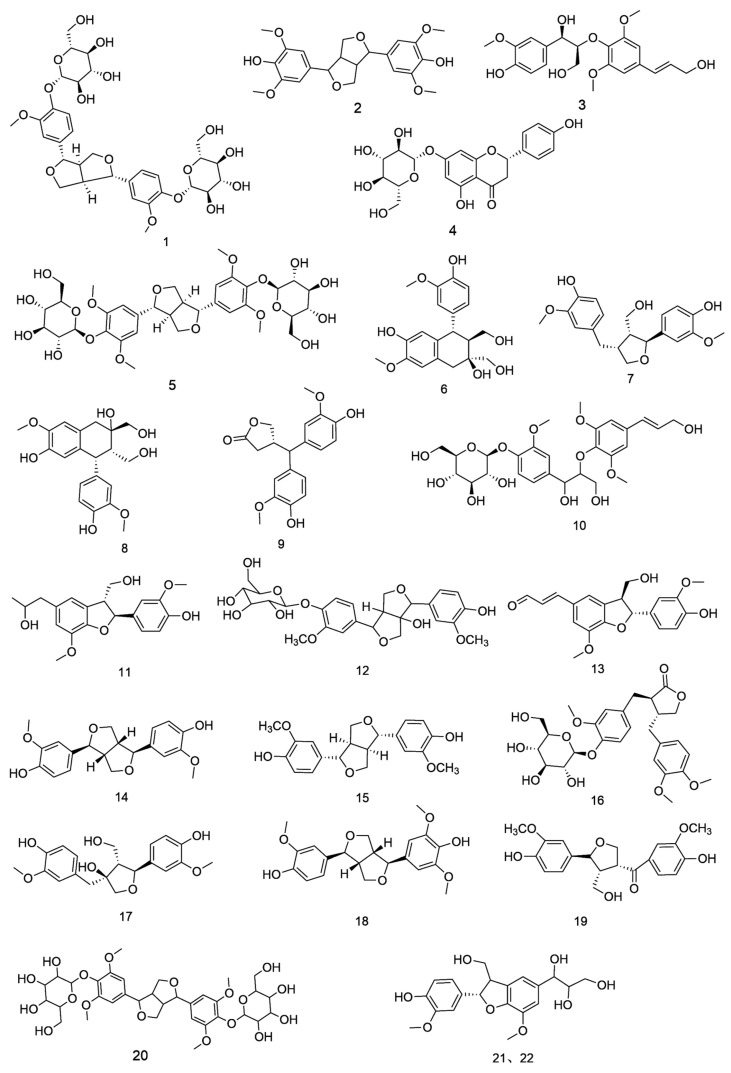
Chemical structures of lignans compounds in *E. ulmoides*.

Lignans, natural products derived from oxidative polymerization of phenylpropanoids, exhibit pharmacological properties including anti-osteoporotic [8], blood metabolism-regulating [9], and antihypertensive effects [10,11]. Fifty-two lignan compounds have been identified in *E. ulmoides*, primarily categorized as bisepoxylignans, monoepoxylignans, neolignans, and cyclolignans [12]. In the quality control of Eucommia ulmoides bark, pinoresinol diglucoside, which is the active constituent responsible for antihypertensive effects, is specified in the Chinese Pharmacopoeia (2020 edition) as a quality control marker with a required minimum content of 0.10%. The highest lignan concentration was detected in the bark, with lower levels present in fruits and leaves. As the most extensively studied phytochemicals in *E. ulmoides* with well-characterized structures, lignans underlie its antioxidant, hypotensive, and immunomodulatory activities (Table 1 and Figure 3) [13,14]. However, most of these activities have been demonstrated primarily in cellular or animal models, and direct clinical evidence supporting lignans as independent therapeutic agents remains limited. Therefore, while lignans represent important bioactive markers for quality control, their contribution to clinical efficacy likely depends on multi-component interactions rather than single-compound effects.

### 3.2. Iridoids

**Figure 4:**
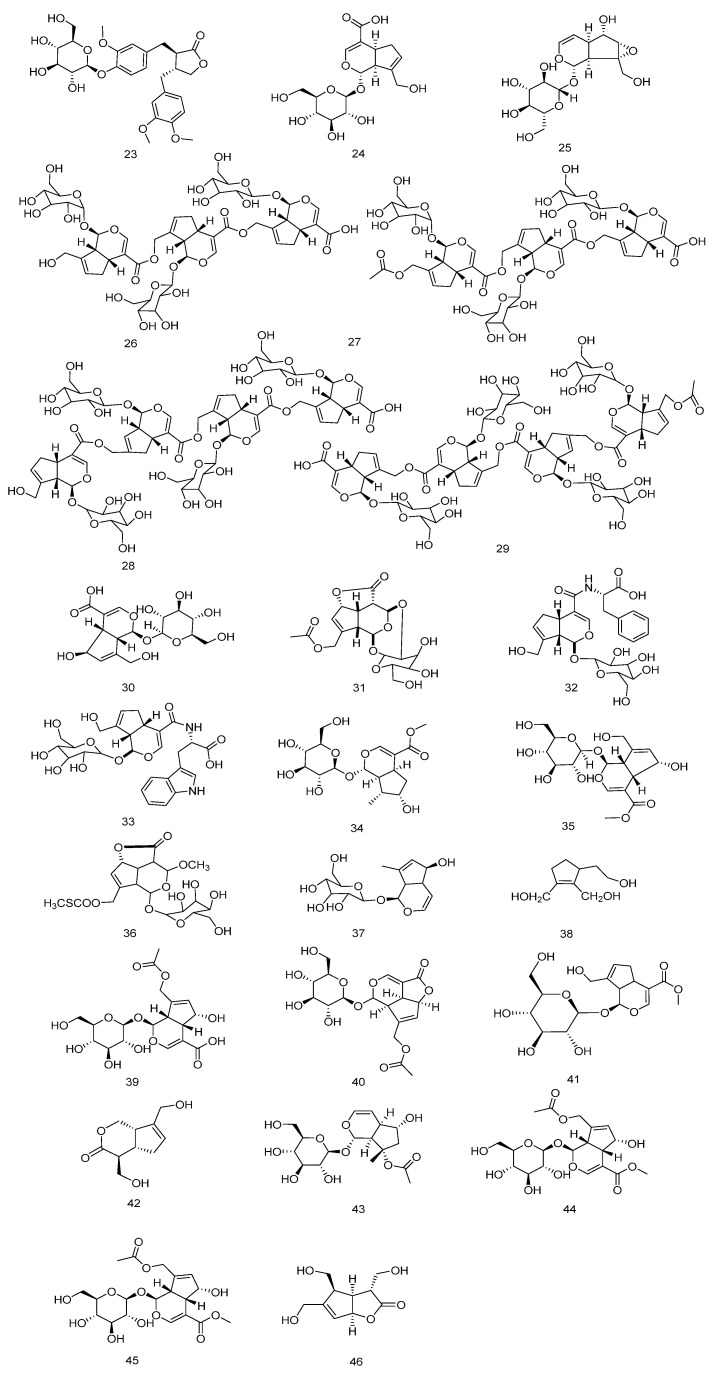
Chemical structures of Iridoids compounds in *E. ulmoides*.

Iridoids are acetal derivatives of iridodial, classified as monoterpenoids characterized by an iridoid alcohol backbone typically stabilized through glycosylation to form iridoid glycosides, featuring hemiactal and cyclopentane ring structures [4,20]. The antitumor, antidiabetic (including complication-alleviating), antiarrhythmic, antispasmodic, immunostimulatory, hepatoprotective, and anti-inflammatory properties of *E. ulmoides* are closely associated with its iridoid constituents. The bark and leaves contain abundant iridoids (~5.07 mg/g) [21], primarily genipin, geniposide, geniposidic acid, and aucubin (Table 2 and Figure 4).

### 3.3. Phenylpropanoids

**Figure 5:**
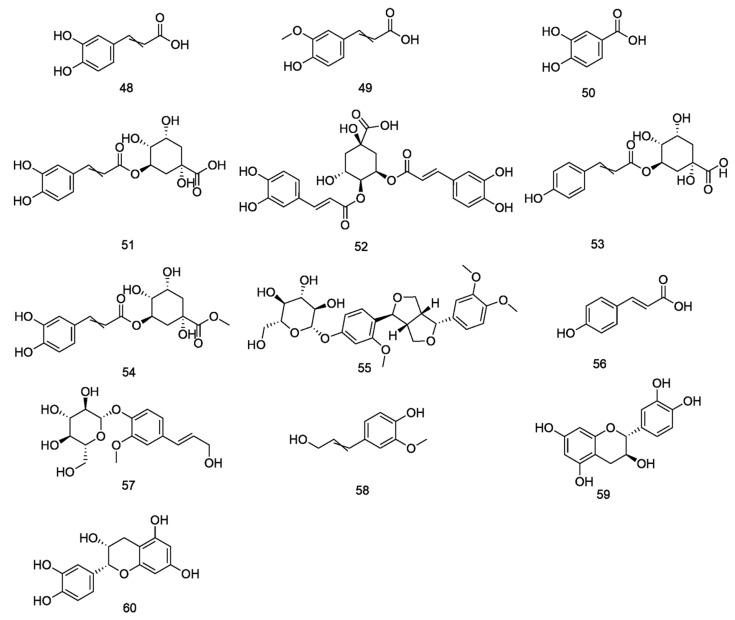
Chemical structures of Phenylpropanoids compounds in *E. ulmoides*.

Phenylpropanoids in *E. ulmoides*-precursors to lignan biosynthesis-included caffeic acid, coniferyl alcohol, guaiacylglycerol, coniferin, syringin, chlorogenic acid, methyl chlorogenate, vanillic acid, and coumaroyl glycosides, distributed in the bark, leaves, and rhizomes. Chlorogenic acid is a critical quality control marker for *E. ulmoides* leaves, with content specified to exceed 0.08% in the Chinese Pharmacopoeia [2]. These compounds exhibited antihypertensive, hypolipidemic, immunostimulatory, antibacterial, and anticancer activities. Chlorogenic acid has been observed to reduce blood pressure in spontaneously hypertensive rats (Table 3 and Figure 5) [24]. Nevertheless, these findings are derived from animal models, and their relevance to human blood pressure regulation requires further clinical validation.

### 3.4. Flavonoids

**Figure 6:**
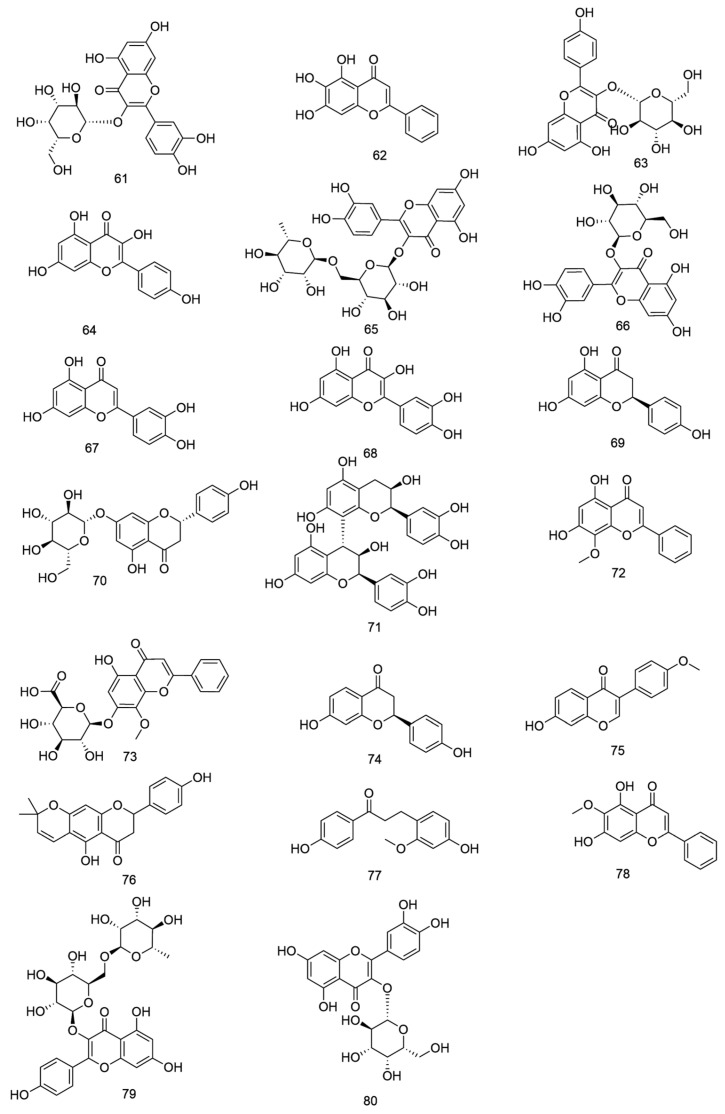
Chemical structures of Flavonoids compounds in *E. ulmoides*.

Flavonoids are compounds formed by two phenolic hydroxyl-bearing benzene rings linked via a central three-carbon chain, predominantly constituting derivatives of chromone. As one of the primary active constituents in *E. ulmoides*, flavonoids were primarily distributed in male flowers and leaves. Key flavonoid components included quercetin, rutin, kaempferol, astragalin, and hyperoside [28,29], which exhibited antioxidant, cardiovascular-regulatory, immunoenhancing, antibacterial, expectorant, and antitussive properties (Table 4 and Figure 6) [30,31].

### 3.5. EUG

**Figure 7:**
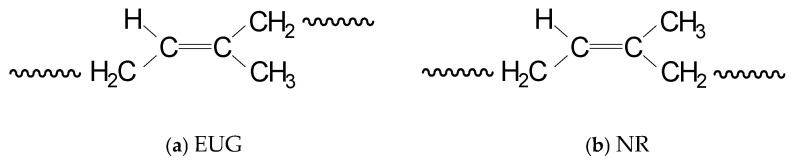
Structure of Gutta-percha and natural rubber.

EUG constitutes a renewable natural rubber resource, primarily composed of a high polymer of trans-isoprene. Its molecular structure represents an isomer of natural rubber (Figure 7). As China’s most promising alternative and supplemental natural rubber resource, the development of the EUG industry not only addresses domestic the development of the EUG industry, but also establishes a new global rubber industry paradigm centered on China’s innovative EUG products. Owing to its combination of low yield strength, rigidity, and toughness, EUG is suitable for manufacturing orthopedic fixation splints [33]. Concurrently, shape-memory materials based on EUG represent a research focus in this field [34].

Studies indicated that EUG serves both as a natural rubber supplement and as a synergist for enhancing composite properties. For instance, EUG/natural rubber composites with optimized ratios could modulate stress–strain curve behavior, improved application characteristics, and exhibited dual rubber–plastic properties, yielding superior functional materials with broader utility [35]. Elasticity improvement was achieved by Yan Ruifang through pervulcanization, creating novel polymeric materials with rubber–plastic duality [36]. High-performance EMI shielding biocomposites can be fabricated using crystalline EUG as the matrix and CNT/GNP hybrids as conductive fillers, demonstrating attractive mechanical properties and high thermal stability [37]. Recent research reveals that composite films prepared from lignin and EUG exhibit exceptional UV-shielding capacity, enhanced thermal stability, improved mechanical properties, and superior aging resistance, indicating potential applications in sustainable packaging and agricultural coatings [38]. Although EUG itself does not function as a pharmacologically active compound, its unique physicochemical properties expand the application scope of *E. ulmoides* beyond bioactivity-driven uses toward biomedical materials and health-related technologies.

### 3.6. Steroids and Other Terpenoids

**Figure 8:**
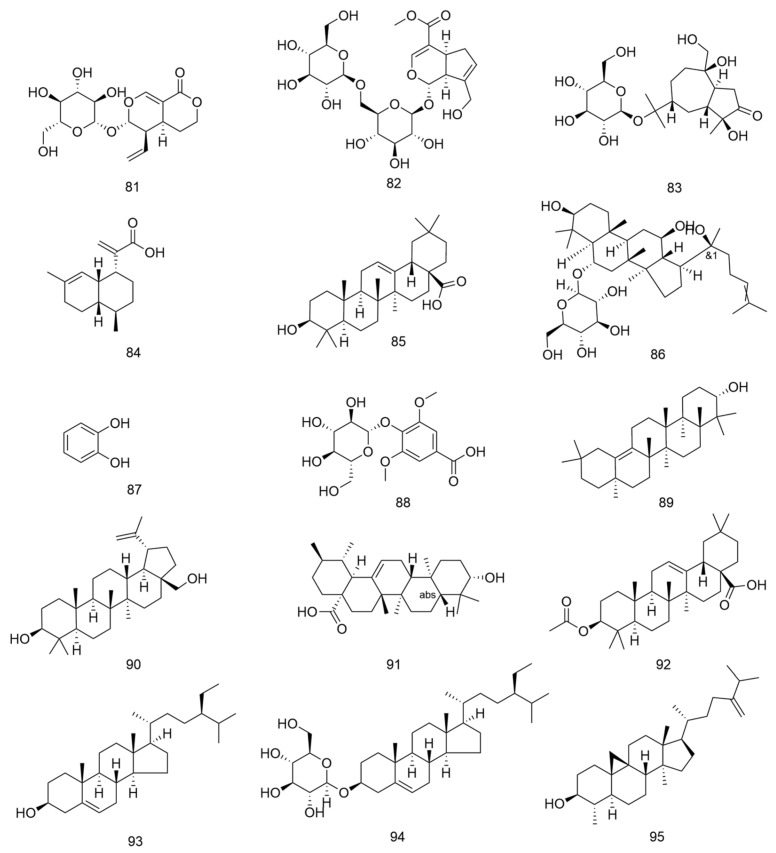
Chemical structures of Terpenoids compounds in *E. ulmoide*.

Steroids exhibit diverse structures and extensive applications in pharmaceuticals. Common biologically active steroids include cholesterol, bile acids, sex hormones, vitamin D, and certain antibiotics. They possess anti-inflammatory, antitoxic, antiallergic, and antishock properties, serving as critical adjunct therapies for collagen diseases, anaphylactic shock, Addison’s disease, breast cancer, and prostate cancer (Table 5 and Figure 8) [39].

The terpenoid composition in *E. ulmoides* is complex, encompassing not only the primary active iridoids but also significant quantities of other terpenoids such as triterpenes. Triterpenes constitute a class of terpenoids polymerized from six isoprene units, featuring a fundamental skeleton of 30 carbon atoms. They existed in plants either in free form or as glycosides/esters, demonstrating diverse biochemical activities.

### 3.7. Others

Additional components in *E. ulmoides* include polysaccharides, amino acids, vitamins, and minerals. Anticarcinogenic effects of *E. ulmoides* polysaccharides have been demonstrated through scavenging nitrite (NO_2_^−^), a precursor of N-nitrosamine synthesis [41]. Exercise-induced fatigue in mice could be alleviated by these polysaccharides via regulation of glucose metabolism and protein-sparing effects [42]. An antifungal protein isolated from the bark has been shown to effectively inhibit fungal growth, exhibiting significant application potential in pharmaceuticals, food safety, and environmental protection.

In conclusion, the chemical constituents of *E. ulmoides* are diverse, mainly consisting of lignans, iridoids, flavonoids, and other compounds. These constituents show a broad range of pharmacological activities, including antioxidant, anti-inflammatory, Liver and kidney protection, cardiovascular protective, neuroprotective, and anti-fatigue effects. Overall, these findings support the medicinal value of *E. ulmoides*.

## 4. Research on Chemical Composition and Pharmacological Functions of *E. ulmoides* Botanical Parts

**Figure 9:**
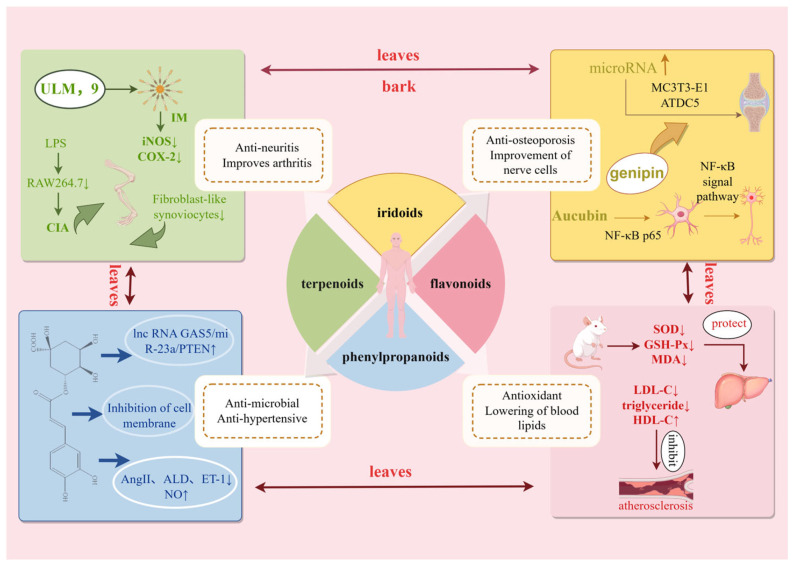
*E. ulmoides* pharmacological mechanism of action.

Traditional utilization of *E. ulmoides* primarily focused on the medicinal use of its bark, which remains the predominant resource in TCM clinical practice. With advancing research and industrial technology, the value of other botanical parts—including leaves, male flowers, fruits, and stems—has gained increasing attention. Consequently, the whole-plant resources of *E. ulmoides* have entered practical applications (Figure 9).

### 4.1. E. ulmoides Bark

#### 4.1.1. Chemical Composition of *E. ulmoides* Bark

Studies revealed similar chemical profiles across *E. ulmoides* plant parts, though with quantitative variations [4]. Key bioactive constituents in the bark encompass lignans, iridoids, phenylpropanoids, flavonoids, and polysaccharides [5], supplemented by EUG, amino acids, antifungal proteins, and trace minerals (e.g., Ca, Fe). The bark contains all characteristic compound classes of *E. ulmoides*, with lignans and EUG being predominant. As the principal component of *E. ulmoides* resources, the bark represents the most extensively utilized segment in current resource development.

#### 4.1.2. Pharmacology and Health Functions of *E. ulmoides* Bark

**Figure 10:**
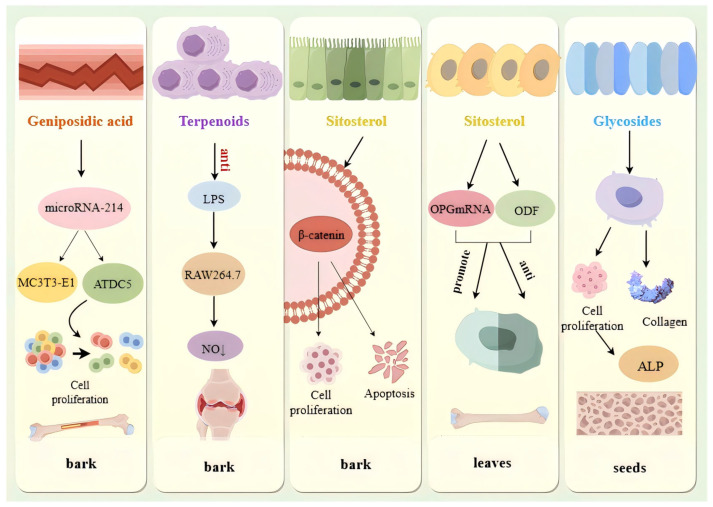
Antiosteoporosis mechanisms of *E. ulmoides*.

*E. ulmoides* is a prized traditional Chinese medicinal herb with a documented history dating back to the Shen Nong Ben Cao Jing from the Eastern Han Dynasty. As stipulated by the pharmacopoeia of the People’s Republic of China [2], the official medicinal part is the bark. Pharmacological actions of *E. ulmoides* bark primarily include antihypertensive [43], hepatoprotective [44], anti-inflammatory, anti-osteoporotic, neuroprotective, and antioxidant effects [4]. The antifungal protein isolated from the bark by Liu et al. [45] effectively inhibited fungal growth, indicating significant application potential in pharmaceuticals, food safety, and environmental protection. Recent animal studies have revealed potential efficacy against postpartum depression through suppression of hypothalamic–pituitary–adrenal axis hyperactivation, downregulation of corticotropin-releasing factor receptor 2, inhibition of voltage-dependent anion channel 1, and reduced neuronal apoptosis [46]. Neuroprotective effects observed in experimental models against optic neuropathy are exerted via AMPK signaling activation that mitigates oxidative stress [47]. In KGN cell lines used pregnenolone as substrate, iridoids were investigated for progesterone, testosterone, and estradiol modulation by Zuo et al. [48]. Elevated genipin concentrations significantly upregulated the expression of 3β-hydroxysteroid dehydrogenase, CYP17A1, and 17β-hydroxysteroid dehydrogenase, thereby stimulating testosterone and estradiol synthesis. Renal oxidative stress in diabetic nephropathy mice was alleviated by *E. ulmoides* flavonoids through mediation of the Nrf2/HO-1 pathway, thereby reducing fasting blood glucose and improving renal function [49]. Monomers such as quercetin, rutin, and hyperoside have been shown to promote osteogenic differentiation [50]. Astragalin and baicalein significantly stimulate MC3T3-E1 Subclone 14 osteoblast proliferation and maturation, counteracting osteoporosis-induced bone loss [51]. Additionally, quercetin exerts antithrombotic and antihypertensive effects through inhibition of platelet lipoxygenase and cyclooxygenase enzymes [52]. Anti-inflammatory effects were demonstrated by Tang et al. [53] through inhibition of NO release in lipopolysaccharide-induced RAW264.7 cells, thereby alleviating joint inflammation and bone destruction in collagen-induced arthritis rats. Osteoclastogenesis inhibition and bone protection were achieved through activation of the β-catenin signaling pathway, effectively ameliorating postmenopausal osteoporosis [54] (Figure 10). Their natural origin and low toxicity provide a valuable direction for developing new drugs to regulate bone metabolism, especially in an aging society, which has significant clinical relevance. Overall, pharmacological evidence for *E. ulmoides* bark is relatively robust at the preclinical level, whereas clinical validation relies largely on traditional usage and compound formulations rather than standardized monotherapies.

**Figure 11:**
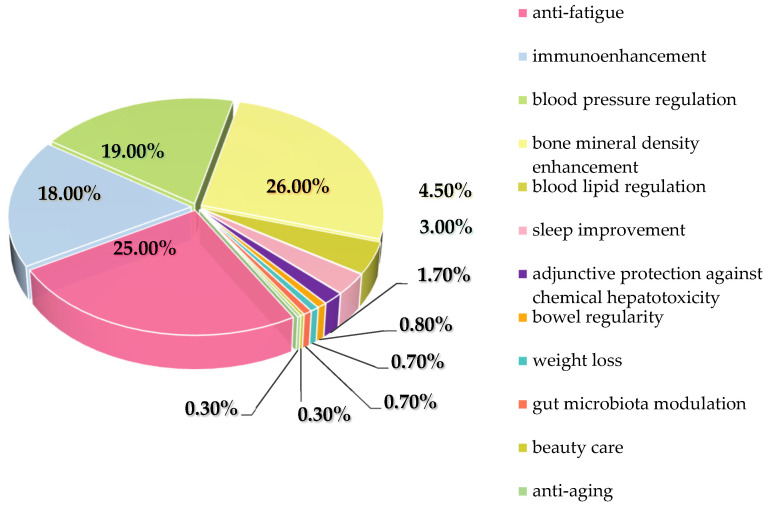
Functional distribution of health food related to *E. ulmoides* bark.

Widely utilized in health foods as extract ingredients, According to the National Medical Products Administration (NMPA) (excluding leaf-based products), as of June 2025, a total of 250 registered health food products using *E. ulmoides* bark as the raw material have been documented. The claimed health benefits of these products include alleviation of physical fatigue, enhancement of immune function, assistance in lowering blood pressure and blood lipid levels, increase in bone mineral density, improvement of sleep quality, and protection against chemical liver injury. Specific data are presented in Figure 11.

#### 4.1.3. Comprehensive Utilization of the Bark

*E. ulmoides* bark served as an additive in animal feeds to enhance product quality or prevent specific diseases. Incorporation into fish feed could improve growth performance, elevate muscle collagen content, and enhance fish quality [55,56]. Supplementation with 5% bark powder in poultry diets boosts laying performance, augments antioxidant capacity, and modulates gut microbiota in hens. Research demonstrated that incorporating *E. ulmoides* into feed inhibits aspergillus flavus growth, spore germination, and toxin synthesis by compromising cell wall integrity, reducing energy metabolism, and modulating toxin synthesis gene expression, thereby effectively mitigating aflatoxin contamination in feed [57].

Advancement in clean energy technologies have expanded industrial applications. Yang [58] utilized post-extraction bark residues to synthesize biomass-derived porous carbon via carbonization and activation processes. This material demonstrated superior electrochemical performance as a lithium–sulfur battery cathode. Within high-value utilization strategies, EUG constitutes a critical component. Lei compared extraction techniques [59], establishing that subcritical ultrasound-assisted extraction outperforms conventional methods, enabling scalable EUG production while advancing green and sustainable resource utilization. Novel wound dressings fabricated from *E. ulmoides* oligomeric gum and carboxymethyl chitosan significantly reduced infection incidence by establishing optimal sterile environments, thereby mitigating risks of wound deterioration and infection-related complications [60]. Despite these developments, non-pharmaceutical applications remain limited. Current use as a plant-based feed additive fails to achieve high-value utilization. Future research should intensify exploration of multifunctional applications to maximize economic potential.

### 4.2. E. ulmoides Leaves

#### 4.2.1. Chemical Composition of the Leaves

The chemical composition of *E. ulmoides* leaves resembles that of the bark, though each possesses unique constituents with quantitative and qualitative variations in shared compounds. Geniposidic acid, geniposide, and pinoresinol diglucoside are more abundant in the bark, whereas chlorogenic acid predominates in leaves. Studies indicated chlorogenic acid exerts antibacterial effects and modulates blood lipid/glucose levels, suggesting leaves substitution for bark in disease management due to its higher yield and accessibility [61]. Additionally, leaves serve as the primary reservoir for micronutrients including polysaccharides, amino acids, proteins, peptides, vitamins, and minerals.

#### 4.2.2. Pharmacological and Health Functions of the Leaves

According to the Chinese Pharmacopoeia [2], *E. ulmoides* leaves are mildly pungent and warm, acting on the liver and kidney meridians to tonify these organs and strengthen bones/tendons. The leaves have been shown to possess similar compositions and pharmacological activities to the bark [62], primarily including antihypertensive, hypoglycemic, hypolipidemic, anti-fatigue, antioxidant, and antitumor effects. Endothelial dysfunction is prevented both in vivo and in vitro through modulation of the Nrf2/HO-1 signaling pathway [63]. Significant neuroprotection is also achieved by enhanced expression of HO-1, NAD(P)H quinone oxidoreductase 1, and catalase proteins, along with elevated superoxide dismutase and glutathione peroxidase activities through regulation of the PI3K/AKT/GSK-3β/Nrf2 axis [64]. Thirteen anti-neuroinflammatory compounds were isolated from *E. ulmoides* by Han et al. [65], with ursane-type C29 triterpenoid significantly suppressed proinflammatory mediators while down regulated inducible nitric oxide synthase and cyclooxygenase-2 expression, indicating therapeutic potential for neurodegenerative diseases. β-sitosterol extracted from leaves by Zeng et al. [66] stimulated OPG mRNA expression while suppressing ODF expression, directly promoting osteogenesis.

**Figure 12:**
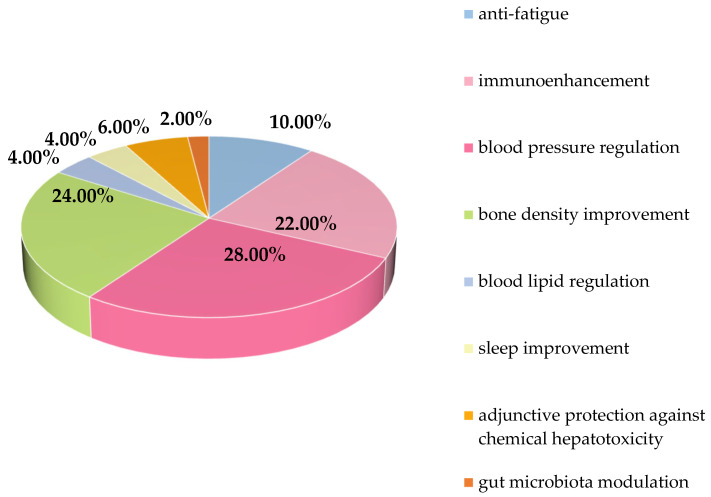
Functional distribution of health food related to *E. ulmoides* leaves.

As a medicinal-food homologous resource, *E. ulmoides* leaves are widely used in health foods. The NMPA database documents 43 registered health products containing *E. ulmoides* leaves, collectively demonstrating seven health functions, with 33 products exhibiting a single function and 8 products demonstrating dual functions. (Figure 12) Beyond overlapping benefits with bark-derived products, leaf formulations uniquely provide auxiliary protection against chemical hepatotoxicity Guo et al. [67] revealed potential hepatoprotective mechanisms involving modulation of vitamin A-related pathways (retinoic acid) and antioxidant activity. Zhang [68] established that suppression of TLR-4/NF-κB signaling through reducing HMGB1 secretion and expression mitigates sterile inflammation in hepatic ischemia–reperfusion injury. Aucubin in leaves may confer liver protection by inhibiting mitochondrial damage-mediated apoptotic pathways [69,70], while chlorogenic acid exhibits comparable hepatoprotective efficacy. Total flavonoids and polysaccharides demonstrated protective effects against CCl4-induced acute liver injury and hepatic fibrosis in experimental models [71,72].

Overall, *E. ulmoides* leaves exhibit notable therapeutic and nutritional value, as evidenced by both traditional medicinal use and modern experimental studies. As a material with dual use as food and medicine, *E. ulmoides* leaves have been widely incorporated into health products and functional foods, reflecting their practical applicability and favorable safety profile. Collectively, current findings highlight the versatility of *E. ulmoides* leaves and emphasize their relevance for further exploration in disease prevention, health promotion, and functional food development.

#### 4.2.3. Comprehensive Utilization of the Leaves

Currently, *E. ulmoides* leaves have been developed into health food products including *E. ulmoides* tea [73], vinegar [74], wine, and jelly, along with daily necessities like toothpaste. Renn et al. [75] innovatively incorporated leaves as functional additives into glutinous rice (Semen Oryzae Glutinosae) fermentation, developing sweet rice wine with potential health benefits including blood glucose reduction, blood pressure regulation, and lipid metabolism modulation. *E. ulmoides* leaves-derived vinegar has been proved to enhance overall antioxidant capacity [76]. Leaf extracts are widely utilized as phytogenic feed additives in aquatic, poultry, and livestock diets (studies indicated that dietary supplementation with *E. ulmoides* leaves or extracts increases unsaturated fatty acid content in muscle tissues, and optimizes egg quality in laying poultry by elevating egg white protein and yolk α-linolenic acid while reducing cholesterol and crude fat content) [77]. When added to fish feed, *E. ulmoides* leaves reduce lipid deposition, elevate muscle collagen content, and improve sensory quality [55,78]. while enhancing meat quality through promoted protein synthesis [79]. Additionally, supplementation reduced livestock disease susceptibility, potentially via immunomodulatory mechanisms. The flavonoid components improved growth performance and intestinal morphology in weaned piglets, reduced coliform colonization and diarrhea incidence, suggesting potential as antibiotic growth promoter alternatives [80]. Jelly developed by Li Zhiying [81] protected against acute alcohol-induced organ damage in mice, while toothpaste formulations showed antibacterial, anti-inflammatory, and analgesic effects against oral diseases [82]. Despite these advances, food and cosmetic applications remain underexplored. Future efforts should expand innovative utilization in these sectors to maximize resource value and economic returns.

### 4.3. Male Flowers

#### 4.3.1. Chemical Composition

*E. ulmoides* is a dioecious plant, with male flowers containing diverse nutritional and functional components including lignans, phenylpropanoids, iridoids, flavonoids, amino acids, polysaccharides, EUG, vitamins, and minerals [83]. Comparative analysis with bark and leaves reveals significant compositional differences in chlorogenic acid, cryptochlorogenic acid, geniposidic acid, quercetin, aucubin, rutin, geniposide, and pinoresinol diglucoside, indicating distinct phytochemical profiles across plant parts [84].

#### 4.3.2. Pharmacological and Health Functions of Male Flowers

*E. ulmoides* male flowers represent a precious medicinal resource with longstanding therapeutic value. Current pharmacological evidence for male flowers is primarily derived from animal models and in vitro studies. Flavonoids and polysaccharides have been demonstrated by Sun Lanping et al. to confer antioxidant properties [30], while pharmacological functions encompassed sedation, hypnotic effects, anti-aging, anti-inflammatory, analgesic, antibacterial, and immunomodulatory activities. Chen Xiaojun [85] found that high-dose aqueous extracts of male flowers show superior efficacy in anti-inflammatory, analgesic, antibacterial, and immunomodulatory activities compared to bark or leaf extracts. Guo Yangjing [86] reported significant reduction in malondialdehyde levels alongside enhanced superoxide dismutase activity and total antioxidant capacity in murine skin tissues, indicating anti-aging effects. Iridoids further increased collagen fiber content and synthesis, synergizing with flavonoids to exert potent anti-photoaging activity. Wang Jianying [87] identified mechanisms involving suppression of Th2 cytokines, restoration of Th1/Th2 balance, downregulation of Th17 cells, inhibition of inflammatory chemotaxis and matrix metalloproteinase activity, and reduced IgE production—collectively alleviating airway inflammation and improving pulmonary pathology. Additionally, components such as rutin and geniposidic acid modulated the CYP11A1 and 17β-HSD signaling pathways to regulate testosterone secretion, conferring anti-fatigue effects. Additionally, four compounds were found to markedly inhibit proliferation of rat fibroblast-like synoviocytes, demonstrating anti-arthritic activity [88].

In summary, *E. ulmoides* male flowers exhibit a broad spectrum of bioactivities, and their multifaceted pharmacological effects suggest considerable potential as a valuable medicinal resource. Nevertheless, the paucity of well-designed clinical studies precludes definitive conclusions regarding their efficacy and safety in humans, highlighting the need for further translational research and rigorous clinical investigations to support their rational development and practical application.

#### 4.3.3. Comprehensive Utilization of Male Flowers

According to Announcement No. 6 (2014) of the National Health Commission of China, *E. ulmoides* male flowers were approved as a novel food ingredient. Their richness in flavonoids, essential amino acids, vitamin C, and trace mineral elements enables broad applications in meal replacements, tea beverages, and medicinal pollens. Zhong Xueting et al. demonstrated that meal replacement powders supplemented with male flowers exhibit lower glycemic index, enhancing suitability for obese individuals and those with glucose metabolism disorders. As of July 2025, 27 invention patents for *E. ulmoides* male flowers tea have been granted. Han Weijuan [89] and Du Hongyan [90] developed formulations containing abundant flavonoids with potent Fe^2+^/Cu^2+^ chelating capacity and significant antioxidant activity. Additionally, these teas demonstrate efficacy in preventing hyperlipidemia [91], counteracting hypertension [92], and conferring hepatoprotection [93]. Noodles incorporating male flowers pollen retained antioxidant, antibacterial, and anti-inflammatory properties while exhibiting a pleasing color and fragrance [94].

### 4.4. Seeds

#### 4.4.1. Chemical Composition

*E. ulmoides* seeds, termed “winged fruits” due to their insect-wing-like appearance, consist of kernels and pericarps. Kernels are rich in unsaturated fatty acids, proteins, amino acids, polysaccharides, vitamins, and bioactive aucubin [95]. Pericarps primarily contain cellulose, lignin, and EUG, with rubber content significantly higher than in bark or leaves. Designated as a novel food ingredient per Announcement No. 12 (2009) of China’s Ministry of Health, *E. ulmoides* seed oil was standardized for properties, production methods, and fatty acid composition. Its fatty acid profile is dominated by ALA (≥45%), followed by OA (≥13%), LA (≥10%), PA (≥6%), and SA (≥2%) (Table 6).

#### 4.4.2. Pharmacological and Health Functions of the Seeds

Li Sen [96] demonstrated that the seeds glycosides directly target osteoblasts, stimulating proliferation and enhancing activity through elevated alkaline phosphatase levels while promoting collagen synthesis to augment bone strength, thereby exerting anti-osteoporotic effects. Additionally, these glycosides might act directly on the reproductive system, significantly increasing indices of the prostate, seminal vesicles, adrenal glands, and testes to enhance reproductive function. Song Linqi [97] found that *E. ulmoides* seed glycosides markedly reduce xylene-induced ear edema in mice and carrageenan-induced paw swelling in rats, significantly prolong thermal pain threshold time, decrease acetic acid-induced writhing responses, and extend photo-electric tail-flick latency, indicating substantial anti-inflammatory and analgesic effects. Miao Jingjing [98] established that seed meal extracts enhance activity of peritoneal macrophages and splenic lymphocytes in postmenopausal osteoporosis rats with immune dysfunction, modulating immune surveillance to restore systemic balance.

Rich in unsaturated fatty acids with α-linolenic acid exceeding 60%, *E. ulmoides* seed oil exhibited lipid-lowering, antiplatelet aggregation, antitumor, immunostimulatory, neurodevelopmental, and cardioprotective properties [99]. Intervention studies indicated amelioration of high-fat diet-induced metabolic dysregulation in mice, improving insulin resistance and reducing hepatic lipid droplets. Potential mechanisms involved modulation of gut microbiota (elevated Bacteroides and Lactobacillus abundance) and bile salt hydrolase-mediated regulation of glycine/taurine-conjugated bile acids to normalize lipid metabolism [100].

In summary, *E. ulmoides* seeds and their derived products demonstrate diverse and complementary biological activities. Collectively, existing evidence highlights the multifunctional potential of *E. ulmoides* seeds, supporting their further exploration in the prevention and management of metabolic, skeletal, and immune-related disorders.

#### 4.4.3. Comprehensive Utilization of the Seeds

**Figure 13:**
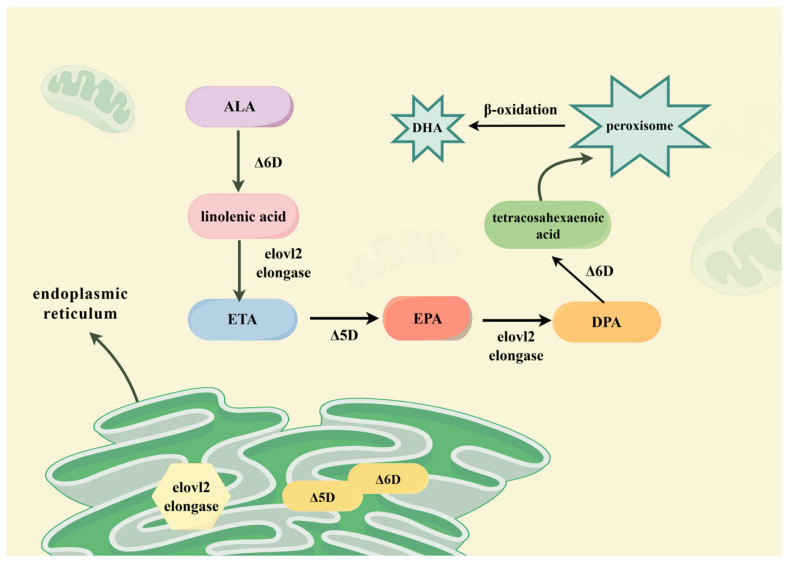
Synthesis of EPA and DHA from ALA metabolism.

*E. ulmoides* seeds are rich in oil, which is approved as a novel food ingredient. Free from poorly digestible substances like erucic acid and behenic acid, the seed oil represents a promising functional resource for food, pharmaceutical, and cosmetic applications [101]. Its utilization in food products continues to expand. The fruit is exceptionally rich in ALA acid (≥60%), a precursor to EPA and DHA [102,103] with demonstrated hypolipidemic, cholesterol-lowering, cardioprotective, developmental, anti-inflammatory, and antioxidant properties [104]. Humans lack ω-3/Δ-15 FAD, making dietary α-linolenic acid essential. Thus, *E. ulmoides* fruit serves as an oil source supplying EPA/DHA precursors or as raw material for EPA/DHA synthesis (Figure 13), enhancing economic value. Wang Xiaoyuan et al. [105] developed ALA-fortified soft candies with seed oil centers, while Niuhan [106] created functional beverages. Seed oil encapsulated in chitosan nanoparticles demonstrates enhanced antibacterial and antioxidant capacities, revealing deeper medicinal potential [107]. Additionally, it is utilized in health foods and pharmaceuticals targeting hyperlipidemia, aging delay, and hyperglycemia [108,109]. The interest in plant-derived interventions for metabolic disorders, including diabetes, has been extensively discussed in recent reviews, which summarize a wide range of botanicals, bioactive compounds, and underlying mechanisms [110]. Within this context, studies on *E. ulmoides* seed oil provide preliminary evidence supporting its metabolic regulatory potential.

*E. ulmoides* seeds contain the highest EUG content among all botanical parts. As a premium renewable rubber resource, its exploitation holds significant importance for national rubber industry development. Xu Zhenchuan [111] utilized crushed seed shells to investigate H_2_O_2_ pretreatment effects on extraction yield, demonstrating that H_2_O_2_ pretreatment significantly enhanced EUG extraction without altering molecular chain structures or chemical composition. Zhang Yue [112] designed and optimized a focused microwave-assisted extraction process, where cellulose removal via microwave treatment prior to petroleum ether extraction substantially improved efficiency. Current application research on seed-derived EUG remains insufficient. Considering abundant oil co-presence, targeted studies on rubber extraction from post-oil-pressing residues could enhance economic value-added benefits.

### 4.5. Other Botanical Parts

Current bark harvesting practices for *E. ulmoides* primarily involve tree felling, generating substantial stem residues that are largely discarded as fuel or timber. However, their use as fuel yields minimal economic return, and their structural instability and processing difficulties limit their applications as timber. The imperative for sustainable utilization of these residues has become an urgent environmental and resource challenge. Recent studies leveraged the high cellulose and lignin content of stems for edible fungus cultivation substrates; for instance, post-bark-harvested trunks can be used to effectively cultivate Auricularia fungi enriched with chlorogenic acid [113]. Discarded branches further served as raw material for nanocellulose production, isolating nanocellulose from *E. ulmoides* cellulose to establish novel pathways for high-value utilization [114]. Future research should prioritize comprehensive investigations into the cellulose and lignan-rich characteristics of stem resources to maximize their valorization potential (Table 7).

## 5. Clinical Research on *E. ulmoides*

In clinical applications, the bark of *E. ulmoides* is mainly used, followed by the leaves. It has been applied in the treatment of orthopedic diseases such as osteoporosis, osteoarthritis, and lumbar disc herniation, as well as hypertension, neurological disorders, and gynecological conditions including threatened abortion and polycystic ovary syndrome. The clinical efficacy of *E. ulmoides* is closely related to its traditional functions of tonifying the liver and kidneys and strengthening tendons and bones.

### 5.1. Orthopedic Diseases

In TCM, bone and joint diseases are generally classified as “Bi syndrome,” which primarily affects the joints and is closely associated with the functions of the liver, spleen, and kidneys. As a representative herb for tonifying the liver and kidneys, *E. ulmoides* exhibits significant therapeutic effects in the prevention and treatment of bone fractures [115], osteoporosis [116], and lumbar disc herniation [117].

The Quan-Du-Zhong capsule, composed of *E. ulmoides* as a single herb, improves joint function and prevents femoral head necrosis by increasing bone mineral density and trabecular number [118]. The classic prescription Qing-E Pill, which uses *E. ulmoides* as the principal ingredient together with *Psoralea corylifolia*, *Juglans regia*, and *Allium sativum*, regulates bone metabolism through its effects of warming and tonifying the liver and kidneys and strengthening muscles and bones. It promotes osteoblast proliferation and exerts estrogen-like activity, thereby improving clinical symptoms and enhancing patients’ quality of life [119]. In addition, *E. ulmoides* often serves as a key herb for tonifying the liver and kidneys in compound formulations, working synergistically with *Taxillus sutchuenensis*, *Achyranthes bidentata*, *Dipsacus asper*, and *Epimedium brevicornu*. It is widely used in clinically approved traditional Chinese patent medicines such as Fufang Duzhong Jian’gu Granules, Duzhong Yaotong Pills, Duhuo Jisheng Decoction [120], and Danlu Tongdu Tablets. However, most clinical evidence in this area is derived from observational studies or small-scale interventions, and high-quality randomized controlled trials are still limited.

### 5.2. Cardiovascular Diseases

In TCM, hypertension is categorized under the patterns of “vertigo” and “headache,” involving primarily the liver, spleen, and kidneys. Clinically, *E. ulmoides* is often combined with Western medicines to treat hypertension, including mild essential hypertension [121], renal hypertension [122], and pregnancy-induced hypertension [123]. In addition to the Quan-Du-Zhong capsule, *E. ulmoides* granules, prepared from both the bark and leaves, can improve endothelial function in patients with hypertensive nephropathy, thereby alleviating vascular constriction and protecting renal function [122].

Professor Tong formulated a prescription composed of *Achyranthes bidentata*, fried *E. ulmoides*, and Taxillus sutchuenensis for the “kidney-deficiency” type of hypertension, achieving significant antihypertensive efficacy and improvement of symptoms such as insomnia, headache, and irritability [124]. The Duzhong Jiangya tablet, which consists of *E. ulmoides*, *Leonurus japonicus*, *Prunella vulgaris*, *Scutellaria baicalensis*, and *Uncaria rhynchophylla*, exerts antihypertensive effects through the regulation of nitric oxide and endothelin balance, enhancement of antioxidant capacity, and promotion of reactive oxygen species clearance [125]. While these studies suggest potential antihypertensive benefits, variability in formulations and outcome measures limits the strength of clinical conclusions.

### 5.3. Neurological Disorders

In TCM, the treatment of neurological disorders emphasizes nourishing the liver and kidneys, clearing the heart, calming the liver, resolving phlegm, and promoting blood circulation. Tian-Zhi granules, which consist of ingredients such as *Gastrodia elata*, *E. ulmoides*, *Uncaria rhynchophylla*, *Taxillus sutchuenensis*, *Haliotis diversicolor*, *Leonurus japonicus*, *Polygonum multiflorum*, *Sophora japonica* flower, and *Gardenia jasminoides*, are effective in alleviating neuropsychiatric symptoms or liver-yang hyperactivity syndrome in patients with Parkinson’s disease. Additionally, this formula enhances cognitive function and mental well-being, showing synergistic effects when used in combination with donepezil [126]. Current evidence mainly supports adjunctive use, and further controlled trials are needed to confirm efficacy.

### 5.4. Tocolytic Effects

*E. ulmoides* exerts tocolytic effects by improving vascular endothelial function, enhancing maternal–fetal immune capacity, supplementing trace elements, strengthening adrenocortical function, and providing antioxidant protection [127]. In clinical practice, it is often combined with spleen-strengthening and stomach-regulating herbs such as *Astragalus mongholicus*, *Dioscorea opposita*, and *Atractylodes macrocephala*. This combination reinforces spleen qi and kidney essence, stabilizes the Chong and Ren meridians, and nourishes the fetus. Current clinical evidence supports the broad therapeutic potential of *E. ulmoides* in orthopedic. However, definitive conclusions are currently limited by a reliance on observational studies and a scarcity of high-quality randomized controlled trials. Future rigorous research is therefore essential to validate these therapeutic claims and support the translational development of *E. ulmoides* in modern medicine and functional foods.

## 6. Prospects for Whole-Plant Resource Utilization

*E. ulmoides*, as a relict plant and traditional medicinal resource unique to China, contains abundant bioactive compounds in its various tissues with broad pharmacological effects. Extensive studies have confirmed that *E. ulmoides* extracts and their monomeric compounds exhibit significant efficacy in lowering blood pressure, regulating glucose and lipid metabolism, anti-inflammatory and antioxidant activities, immune modulation, and promoting bone formation, indicating great potential for the prevention and auxiliary intervention of chronic diseases such as hypertension, diabetes, hyperlipidemia, osteoporosis, and inflammation-related disorders. The material basis for these pharmacological activities primarily includes lignans, iridoids, flavonoids, phenylpropanoids, and polysaccharides. Notably, in addition to the aforementioned small-molecule active compounds, various tissues of *E. ulmoides* are also rich in EUG—a natural polymer that is an isomer of natural rubber, possessing unique dual characteristics of rubber and plastic, shape memory effect, and good biocompatibility. These properties make EUG particularly suitable for applications in biomedical materials, tissue engineering scaffolds, smart responsive materials, and environmentally friendly composites, especially in the context of medical rehabilitation and health-related materials.

Although *E. ulmoides* bark has historically been the most extensively utilized medicinal resource, its traditional harvesting practice—removal of bark following tree felling—raises concerns regarding resource depletion, limited renewability, and ecological sustainability. Continued reliance on bark therefore poses long-term challenges for both environmental conservation and industrial development. By contrast, renewable plant tissues such as leaves, male flowers, and fruits provide a more sustainable and safer utilization pathway. *E. ulmoides* leaves can be harvested on an annual basis without compromising plant vitality and are officially recognized as a medicinal–food homologous resource, supporting their safety for long-term dietary consumption. Similarly, the periodic collection of male flowers and fruits does not interfere with normal growth or reproductive processes, further underscoring their suitability for sustainable exploitation and functional food development. It is particularly important to note that *E. ulmoides* seeds not only contain extremely high levels of EUG in their shells but are also rich in *E. ulmoides* seed oil—a functional oil characterized by α-linolenic acid as its main fatty acid, which possesses various physiological functions such as regulating lipid metabolism and improving insulin resistance, suggesting promising applications in the dietary intervention of cardiovascular and metabolic diseases, including atherosclerosis and type 2 diabetes. This provides an important direction for the comprehensive development of *E. ulmoides* resources. Chemical composition studies have shown that *E. ulmoides* leaves, flowers, fruits, and bark share high similarity in their active component profiles, all containing lignans, iridoid glycosides, flavonoids, chlorogenic acid, and phenolic acids. Consequently, they exhibit common pharmacological activities in areas such as antihypertensive, hypoglycemic, antioxidant, anti-inflammatory, and bone-protective effects, supporting their potential functional application in chronic disease prevention, bone health maintenance, and metabolic health management. Based on the similarity in composition and function, *E. ulmoides* leaves, male flowers, and seed oil have been approved as new food ingredients or medicinal and edible substances in China, providing a regulatory basis for their application in health products. Various products, such as *E. ulmoides* tea, fermented *E. ulmoides* wine, and *E. ulmoides* seed oil soft capsules, have been developed, demonstrating promising prospects and market potential.

Despite significant advancements in characterizing the chemical profile and pharmacological properties of *E. ulmoides*, critical knowledge gaps continue to impede its clinical translation and industrial scalability. While extensive in vitro and in vivo studies underscore its multi-target therapeutic potential, robust clinical evidence remains scarce. Furthermore, standardized toxicological evaluations—particularly regarding long-term, high-dose administration and the safety of bark-derived functional foods—are currently insufficient. These limitations underscore the urgent need for rigorous, systematic research to establish a foundation for evidence-based applications.

Future research should prioritize the clinical validation of standardized extracts and primary bioactive compounds, alongside comprehensive safety assessments to define precise dosage regimens. Concurrently, a comparative functional analysis of renewable tissues (leaves, male flowers, and seeds) is essential to evaluate their potential as sustainable substitutes for bark. Elucidating the synergistic multi-component interactions and structure–function relationships will also be pivotal in providing a scientific rationale for their health-promoting effects.

From a sustainability perspective, prioritizing renewable resources is imperative for future development. *E. ulmoides* leaves, recognized as a “medicinal-food dual-use” resource, offer a safe and sustainable matrix for long-term dietary intervention, while the α-linolenic acid-rich seed oil holds significant promise for cardiovascular and metabolic health. Additionally, advancing green extraction and modification technologies for EUG could catalyze its application in biomedical and eco-friendly materials. Integrating chemical characterization, functional evaluation, and safety assessments will facilitate the transition of *E. ulmoides* into high-value products, contributing to both public health and the global bio-based economy.

In summary, the high-value development and utilization of the entire *E. ulmoides* resource should adhere to the principle of sustainable development, prioritizing the in-depth development and industrial application of renewable parts such as leaves, flowers, and fruits, and constructing a diversified industrial chain covering pharmaceuticals, functional foods, cosmetics, feed, and bio-based materials. By closely linking functional efficacy with disease prevention and health promotion needs, the utilization of *E. ulmoides* bark in the health product sector must be cautiously advanced based on scientific safety evaluations, while resource-saving and environmentally friendly harvesting and processing models should be explored. Through the synergistic advancement of compositional research, technological innovation, and industrial chain integration, *E. ulmoides* resources are expected to become an important component of the regional bioeconomy, achieving harmony among ecological, economic, and social benefits.

## Figures and Tables

**Table 1:** Lignans in *E. ulmoides*.

NO.	Compound	Formula	Part	Reference
1	pinoresinol diglucoside	C_32_H_42_O_16_	leaf/bark/seeds	[15]
2	(+)-syringaresinol	C_22_H_26_O_8_	leaf/bark/seeds	[15]
3	guaiacylglycerol-8-O-4′- (sinapyl aldehyde) ether	C_20_H_24_O_7_	bark	[15]
4	prunin	C_20_H_24_O_7_	bark	[15]
5	syringaresinol-di-O-glucoside	C_34_H_46_O_18_	bark	[15]
6	(+)-Cycloolivil	C_20_H_24_O_7_	bark	[15]
7	lariciresinol	C_22_H_28_O_6_	bark	[16]
8	(+)-epicycloolivil	C_20_H_24_O_7_	bark	[16]
9	noreucol A	C_19_H_20_O_6_	bark	[16]
10	citrusin B	C_27_H_36_O_13_	bark	[16]
11	dihydrodehydrodiconiferyl alcohol	C_20_H_24_O_6_	leaf	[16]
12	forsythialanside E	C_26_H_32_O_12_	leaf	[16]
13	balanophonin	C_34_H_42_O_20_	bark	[17]
14	(+)-pinoresinol	C_10_H_18_O	bark	[17]
15	(+)-epinoresinol	C_20_H_22_O_6_	bark	[17]
16	aectiin	C_27_H_34_O_11_	bark	[17]
17	(-)-olivil	C_20_H_24_O_7_	bark	[17]
18	medioresinol	C_21_H_24_O_7_	bark	[4]
19	vladinolD	C_20_H_22_O_7_	bark	[18]
20	liriodendrin	C_34_H_46_O_18_	bark	[18]
21	erythro-dihydroxydehydro-di-coniferyl alcohol	C_20_H_24_O_8_	Bark/leaf	[19]
22	threo-dihydroxydehydro-di-coniferyl alcohol	C_20_H_24_O_8_	bark	[19]

**Table 2:** Iridoids in *E. ulmoides*.

NO.	Compound	Formula	Part	Reference
23	aucubin	C_15_H_22_O_9_	leaf/bark/flowers/seeds	[15]
24	geniposidic acid	C_16_H_22_O_10_	leaf/bark/flowers/seeds	[15]
25	catalpol	C_15_H_22_O_10_	leaf/bark/flowers/seeds	[15]
26	ulmoidoside A	C_48_H_62_O_28_	seed	[15]
27	ulmoidoside B	C_50_H_64_O_29_	seeds	[15]
28	ulmoidoside C	C_64_H_82_O_37_	seeds	[15]
29	ulmoidoside D	C_66_H_84_O_38_	seeds	[15]
30	deacetylasperulosidic acid	C_16_H_22_O_11_	leaf	[15]
31	eucomosides A	C_18_H_22_O_11_	leaf	[22]
32	eucomosides B	C_25_H_31_NO_11_	leaf	[22]
33	eucomosides C	C_27_H_32_N_2_O_11_	leaf	[22]
34	loganin	C_17_H_26_O_10_	leaf	[16]
35	deacetyl asperulosidic acid methyl ester	C_17_H_24_O_11_	leaf	[16]
36	4-dihydro-3-methoxypaederoside	C_19_H_26_O_12_S	flowers	[16]
37	linaride	C_15_H_22_O_8_	seeds	[16]
38	deoxyeucommiol	C_9_H_16_O_3_	bark	[16]
39	asperulosidic acid	C_18_H_24_O_12_	leaf/flowers	[23]
40	asperuloside	C_18_H_22_O_11_	flowers/leaf	[23]
41	geniposide	C_15_H_18_O_9_	leaf/flowers	[23]
42	gardendiol	C_10_H_14_O_4_	bark	[17]
43	artselaenin	C_15_H_22_O_5_	flowers	[17]
44	ajugoside	C_17_H_26_O_10_	leave	[4]
45	daphylloside	C_20_H_28_O_11_	flowers	[4]
46	borreriagenin	C_10_H_14_O_5_	leaf	[20]

**Table 3:** Phenylpropanoids in *E. ulmoides*.

NO.	Compound	Formula	Part	Reference
48	caffeic acid	C_9_H_8_O_4_	leaf/bark/flowers/seeds	[15]
49	ferulic acid	C_10_H_10_O_4_	leaf/bark/flowers/seeds	[15]
50	protocatechuic acid	C_7_H_6_O_4_	leaf/bark/flowers/seeds	[15]
51	chlorogenic acid	C_16_H_18_O_9_	leaf/bark/flowers/seeds	[15]
52	isochlorogenic acid isomer	C_22_H_28_O_14_	leaf/flowers	[16]
53	3-p-coumaroylquinic acid or isomer	C_16_H_18_O_8_	leaf/flowers/seeds	[16]
54	methyl chlorogenate	C_17_H_20_O_9_	leaf/flowers	[16]
55	forsythin	C_27_H_34_O_11_	leaf	[25]
56	*p*-Coumaric acid	C_9_H_8_O_3_	leaf	[25]
57	coniferin	C_16_H_22_O_8_	bark	[26]
58	coniferol	C_10_H_12_O_3_	stems	[26]
59	catechin	C_15_H_14_O_6_	Bark/leaf	[26]
60	L-Epicatechin	C_15_H_14_O_6_	bark	[27]

**Table 4:** Flavonoids in *E. ulmoides*.

NO.	Compound	Formula	Part	Reference
61	hyperin	C_21_H_20_O_12_	leaf/bark	[15]
62	baicalein	C_15_H_10_O_5_	leaf/flowers/seeds	[15]
63	astragalin	C_21_H_18_O_11_	leaf	[15]
64	kaempferol	C_15_H_10_O_6_	leaf/flowers/seeds	[15]
65	rutin	C_27_H_30_O_16_	leaf/bark/flowers/seeds	[15]
66	isoquercitrin	C_21_H_20_O_12_	leaf/flowers/seeds	[15]
67	luteolin	C_15_H_10_O_6_	leaf/bark/flowers/seeds	[15]
68	quercetin	C_15_H_10_O_7_	leaf/flowers/seeds	[15]
69	naringenin	C_15_H_12_O_5_	flowers	[16]
70	prunin	C_21_H_22_O_10_	flowers	[16]
71	procyanidin B2	C_30_H_26_O_12_	bark	[16]
72	wogonin	C_16_H_12_O_5_	bark	[17]
73	wogonoside	C_22_H_20_O_11_	bark	[17]
74	liquiritigenin	C_15_H_12_O_4_	bark	[25]
75	formononetine	C_16_H_12_O_4_	bark	[5]
76	paratocarpin E	C_20_H_18_O_5_	bark	[5]
77	loureirin C	C_16_H_16_O_4_	bark	[5]
78	oroxylin	C_16_H_12_O_5_	bark	[32]
79	nicotiflorin	C_27_H_30_O_15_	bark	[26]
80	hyperoside	C_21_H_20_O_12_	bark	[26]

**Table 5:** Terpenoids in *E. ulmoides*.

NO.	Compound	Formula	Part	Reference
81	sweroside	C_16_H_22_O_9_	leaf/bark/flowers/seeds	[15]
82	genipin 1-gentiobioside	C_23_H_34_O_15_	leaf/bark/flowers	[15]
83	atractyloside A	C_21_H_36_O_10_	leaf/bark/seeds	[15]
84	artemisinic acid	C_15_H_22_O_2_	leaf/bark/flowers/seeds	[15]
85	oleanolic acid	C_30_H_48_O_3_	leaf/bark/flowers/seeds	[15]
86	ginsenoside Rh1	C_36_H_62_O_9_	bark/seeds	[15]
87	catechol	C_6_H_6_O_2_	leaf	[16]
88	glucosyringic acid	C_15_H_20_O_10_	bark	[16]
89	α-amyrin	C_30_H_50_O	flowers	[16]
90	betulin	C_30_H_50_O_2_	bark/seeds	[16]
91	ursolic acid	C_30_H_48_O_3_	bark/seeds	[16]
92	3-O-acetyl oleanolic acid	C_30_H_48_O_3_	flowers	[16]
93	β-sitosterol	C_29_H_50_O	bark/leaf	[4]
94	daucosterol	C_35_H_60_O_6_	bark/seeds	[26]
95	cycloeucalenol	C_30_H_50_O	bark	[40]

**Table 6:** Indicators and specifications of *E. ulmoides* seed oil as a new food ingredient.

Indicators for New Food Ingredients	Relevant Indicator Specifications	
Nature	Yellowish brown transparent oily liquid	
Production process	Seed removal, separation of shells and kernels, followed by pressing and filtration	
Fatty acid composition (% of total fatty acid content)	ALA(C18:3)	≥45%
	OA (C18:1)	≥13%
	LA (C18:2)	≥10%
	PA (C16:0)	≥6%
	SA (C18:0)	≥2%

**Table 7:** Pharmacological activities and related mechanisms of *E. ulmoides*.

Pharmacological Effects	Materials	Subjects Content	Administration Dosage	Effect/Mechanism	References
**Lower blood pressure**	Lignans	Spontaneously hypertensive rats	150 and 300 mg/kg Twice daily, for 14 days	Increasing NO↑ modulating renin–angiotensin system↑ relaxing vessel↑	[9]
	Chlorogenic acid	Spontaneously hypertensive rats	5 and 20 mg/kg for 8 weeks	Antihypertensive effect↑	[24]
**Bone protection**	Astragalin and baiacalein	Osteoprotegerin	7.5, 40, 80 mg/L for 24 h	Proliferation of MC3T3-E1 subclone 14 osteoblasts↑ expression of OPG and Osterix↑ protein expression of RANKL↓	[51]
	Lignans	Sprague-Dawley rats	20, 40, or 80 mg/kg for 12 weeks	Osteoprotegrin↑ NF-κB ligand expression↓	[8]
	Flavonoids	Bone marrow mesenchymal stem cells	0.08, 0.4, 2, 10, 50 μg/mL for 24 d	Osteogenic differentiation of BMSCs↑	[50]
	Iridoids	Ovarian granulosa cells	10, 22, 50 μM for 24 h	The expression levels of 3β-HSD, CYP17A1, and 17β-HSD↑	[48]
	Seeds glycosides	Osteoporotic mice	55, 110, 220 mg/kg for 12 weeks	Direct action on the reproductive system synthesize collagen↑ bone strength↑	[96]
	β-sitosterol	Osteoblasts and ovarian granulosa cells	0.25 μM for 3 d	OPGmRNA expression↑ secretion of ODF↓	[66]
	β-sitosterol	C57BL/6J mice	5 mg/kg for 8 weeks	Osteoblast differentiation↑ β-catenin signaling pathway↑	[54]
	Seeds extracts	Osteoporosis rats	180 mg/kg/d	The activity of macrophages and lymphocytes in the spleen and abdomen↑ levels of TNF-α, IGF-1 and IL-6↓	[98]
**Liver and kidney protection**	Aucubin	Hepatic ischemia–reperfusion injury rats	1, 5, 10 mg/kg/d	Expression of HMGB1↓ TLR-4/NF-kB signaling pathway↓ the sterile inflammatory response of HIRI↓	[68]
	Chlorogenic acid	Hepatic ischemia–reperfusion injury rats	25, 50, 100 mg/kg/d	Anti-inflammatory and antioxidant effects expression of HMGB1↑	[70]
	Flavonoids	Acute liver injury rats	50, 200 mg/kg for 7 d	The content of SOD and GSH↑ the levels of ALT, AST, and MDA↓	[71]
	Polysaccharide	Hepatic fibrosis rats	35, 70, 140 mg/kg for 8 weeks	The content of ALT, AST, HA, LN, IV-C, GLOB, MDA and Hyp↓ the content of TP, ALB, A/G, SOD, GSH-Px↑ expression of TGF-β1↓	[72]
	Water extracts; alcohol extracts	Hepatic ischemia–reperfusion injury rats	20, 40, 80 mg/kg for 10 d	Degree of liver injury↓ inflammatory response and oxidative stress in liver tissue↓	[44]
**Anti-inflammation**	Seeds glycosides	Paw edema and ear swelling mice	55, 110, 220 mg/kg for 7d	Exhibits anti-inflammatory and analgesic effects	[97]
	Triterpenoid and iridoid	Collagen-induced arthritis mice	60 mg/kg for 2 weeks and 4 weeks	Expression of TNF-α, IL-17, and IL-23↓ proliferation of HFLS-RA↓ RAW264.7 cells release NO↓	[53]
	Alcohol extract of bark and male flower	Asthmatic mice	4 g/kg/d	The production of OVA-Ig E↓ secretion of Th2 cytokines ↓ expression of pro-inflammatory cytokines↓	[87]
**Anti** **-** **depress** **ion**	Bark extracts	Postpartum depressive rats	1.34, 2.68 g/kg for 3 weeks	Activation of the HPA axis↓ expression of CRFR2↓ expression of VDAC1↓	[46]
**Effect on Improving Insulin Resistance**	Seed oil	KK-Ay mice	0.9 mg/g	The content of fasting blood glucose, fasting insulin, total cholesterol, total triglyceride and low-density lipoprotein↓ microbial diversity↑	[100]
**Antioxidant**	Flavonoids	Diabetic nephropathy mice	80, 160 mg/(kg·d)	The content of SOD↑ the content of MDA↓ the expression of Nrf2 and HO-1 protein↑	[49]
	Male flowers extracts	Skin photoaging mice	Topical application	Vitality and capacity in mouse skin tissue↑ accumulation of peroxidation products↓	[86]
**Neuroprotection**	Ulmoidol	BV-2 cells	1, 3, 10 μM	The expression of iNOS and COX-2↓ TLR4/MAPK/NF-κB signaling pathways↓	[65]
	Lignans	Diabetic mice RF/6A cells	10 μg/mL for 2 months, 25, 50, 75 and 100 μM	Regulate oxidative stress response in retinal endothelial cell line, retina, and liver regulation of Nrf2/HO-1 signal transduction pathway	[63]
**Anti-fatigue**	Polysaccharide	Long-term exercise-induced fatigue mice	100, 200 mg/kg	The activity of serum CK and the level of BUN↓ regulates glucose metabolism and conserves protein	[42]

## Data Availability

No new data were created or analyzed in this study.
